# Fingerprinting of hydroxyl radical-attacked polysaccharides by *N*-isopropyl-2-aminoacridone labelling

**DOI:** 10.1042/BJ20140678

**Published:** 2014-09-22

**Authors:** Robert A. M. Vreeburg, Othman B. Airianah, Stephen C. Fry

**Affiliations:** *The Edinburgh Cell Wall Group, Institute of Molecular Plant Sciences, School of Biological Sciences, Daniel Rutherford Building, The King's Buildings, Edinburgh EH9 3JH, U.K.

**Keywords:** electrophoresis (high-voltage), fluorescent labelling, hydroxyl radical, non-enzymic scission, pectin, polysaccharide, 1A, 2A, 2B, 2L, X, fluorescently labelled compounds formed from *in-vitro*^•^OH-attacked pectin, AMAC, 2-aminoacridone, GalA, D-galacturonic acid, GalA–pAMAC, reductive amination product formed when D-galacturonic acid is attached via its C-1 to the amino group of *N*-isopropyl-2-aminoacridone [thus, strictly, a compound of 1-amino-1-deoxy-L-galactonic acid], and likewise for other ‘sugar–pAMAC ‘ abbreviations (see Figures 2 and 5), GlcA, D-glucuronic acid, GulA, D-guluronic acid, *m*_OG_, electrophoretic mobility relative to that of Orange G (*m*_OG_=1.0) and corrected for electro-endo-osmosis by reference to glucose (*m*_OG_=0.0), pAMAC, *N*-isopropyl-2-aminoacridone, pAMAC·GalA, galacturonic acid with one of its non-anomeric–OH groups replaced by pAMAC, and likewise for other ‘pAMAC·sugar’ abbreviations (see Figure 2), PyAW, pyridine/acetic acid/chlorobutanol/water (1:1:0.5:98, v/v/w/v), TalA, D-taluronic acid, UA, an unspecified uronic acid

## Abstract

Hydroxyl radicals (^•^OH) cause non-enzymic scission of polysaccharides in diverse biological systems. Such reactions can be detrimental (e.g. causing rheumatic and arthritic diseases in mammals) or beneficial (e.g. promoting the softening of ripening fruit, and biomass saccharification). Here we present a method for documenting ^•^OH action, based on fluorescent labelling of the oxo groups that are introduced as glycosulose residues when ^•^OH attacks polysaccharides. The method was tested on several polysaccharides, especially pectin, after treatment with Fenton reagents. 2-Aminoacridone plus cyanoborohydride reductively aminated the oxo groups in treated polysaccharides; the product was then reacted with acetone plus cyanoborohydride, forming a stable tertiary amine with the carbohydrate linked to *N*-isopropyl-2-aminoacridone (pAMAC). Digestion of labelled pectin with ‘Driselase’ yielded several fluorescent products which on electrophoresis and HPLC provided a useful ‘fingerprint’ indicating ^•^OH attack. The most diagnostic product was a disaccharide conjugate of the type pAMAC·UA-GalA (UA=unspecified uronic acid), whose UA-GalA bond was Driselase-resistant (product **2A**). **2A** was clearly distinguishable from GalA-GalA–pAMAC (disaccharide labelled at its reducing end), which was digestible to GalA–pAMAC. The methodology is applicable, with appropriate enzymes in place of Driselase, for detecting natural and artificial ^•^OH attack in diverse plant, animal and microbial polysaccharides.

## Abstract 2

Non-enzymic scission of polysaccharides by hydroxyl radicals (^•^OH) may be biologically detrimental or beneficial. We present a ‘fingerprinting’ method for detecting polysaccharides that have been ^•^OH-attacked. The method detects the glycosulose residues introduced by ^•^OH action.

## INTRODUCTION

Polysaccharides, proteoglycans and glycolipids play key structural roles in all kingdoms of life, for example in plant, fungal and bacterial cell walls and in the animal extracellular matrix. Numerous glycanases and lyases act on such carbohydrates, cleaving the backbone by hydrolysis [1] or β-elimination [2], and the action of such enzymes *in vivo* contributes to many different biological processes including plant cell expansion and fruit softening, fungal saprophagy [3] and animal morphogenesis and cartilage metabolism.

In addition to enzyme action, the highly reactive hydroxyl radical (^•^OH) can cause polysaccharide chain scission non-enzymically. When a source of ^•^OH is introduced into polysaccharide solutions, their viscosity decreases rapidly [4], and this and other analytical methods indicate mid-chain polymer cleavage *in vitro* [5–7] and during food processing [8,9]. ^•^OH may be generated in living systems by various mechanisms, especially the Fenton reaction, in which a reduced transition metal ion reacts with H_2_O_2_:

$$\begin{array}{ccc} {\mathrm{Cu}}^{+} & + & H_{2}O_{2}\to {\mathrm{Cu}}^{2+}{+}^{•}\mathrm{OH}+{\mathrm{OH}}^{-}\\ {\mathrm{Fe}}^{2+} & + & H_{2}O_{2}\to {\mathrm{Fe}}^{3+}{+}^{•}\mathrm{OH}+{\mathrm{OH}}^{-} \end{array}$$

[4,5,10–12]. Such radical-mediated scission may either be detrimental, for example leading to rheumatism and arthritis in mammalian joints when hyaluronan is attacked [13–16], or beneficial, for example facilitating rapid plant cell expansion [11], fruit softening [17,18] and seed germination [19], and facilitating the digestion of leaf/wood-litter polysaccharides by saprophytic fungi [20,21].

**Figure 1:**
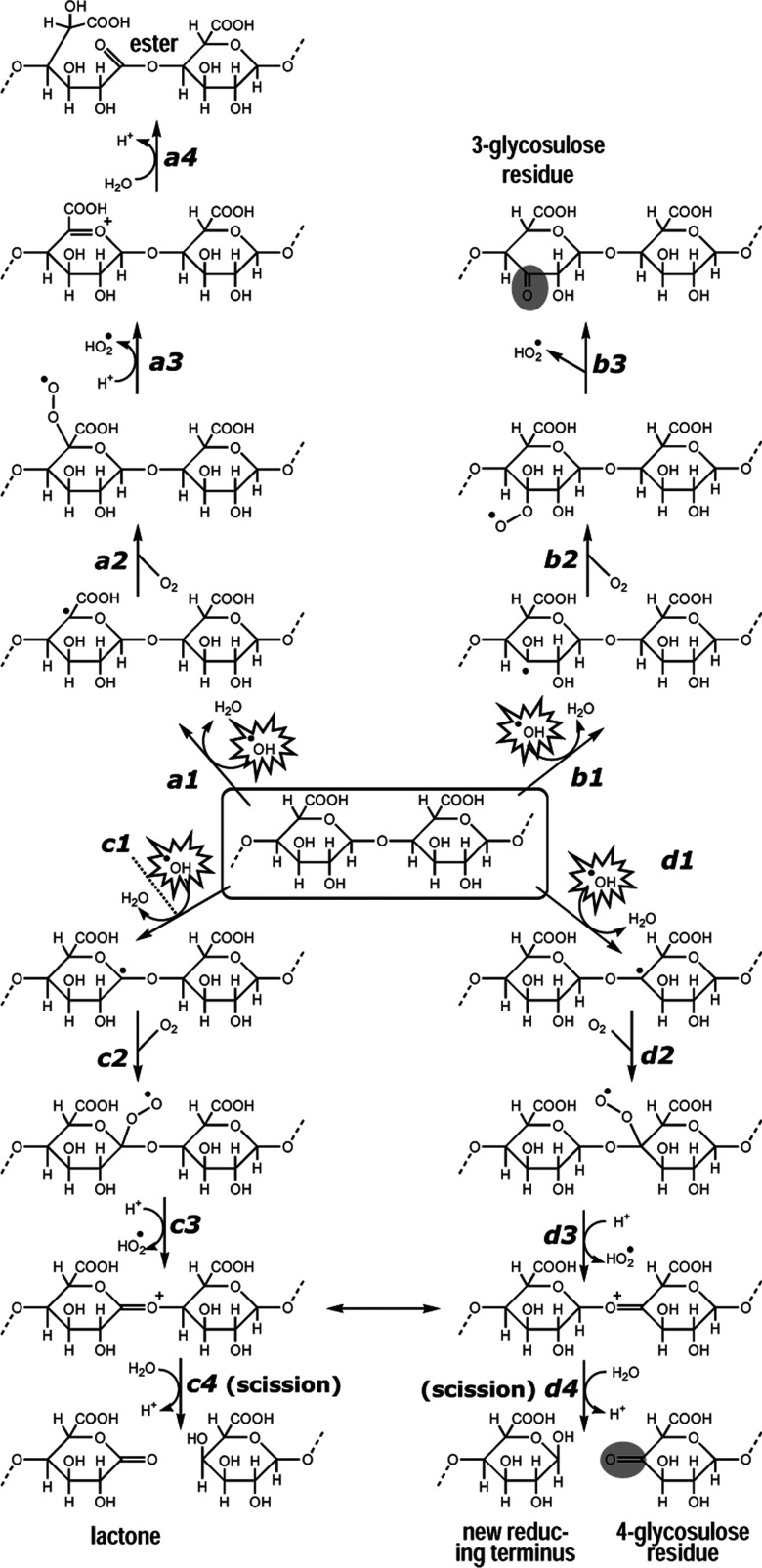
Proposed action of ^•^OH on a polysaccharide chain in aerobic aqueous solution The diagram shows some of the proposed reactions with reference to the fate of homogalacturonan (box in centre, where–––=continuation of polysaccharide chain). It illustrates the predicted reactions occurring after ^•^OH abstracts a hydrogen atom from C-1 (reactions *c1*–*c4*), C-3 (reactions *b1*–*b3*), C-4 (reactions *d1*–*d4*) or C-5 (reactions *a1*–*a4*). Abstraction of hydrogen from C-2 (not shown) is expected to give products directly comparable with those shown for C-3. Adapted from [30,44].

In addition, some polysaccharide-degrading enzymes have recently been found to act by achieving oxidative scission [22,23] in reactions which may be similar to those seen in Fenton systems. The detailed reactions catalysed by such oxidases remain unclear, and limited methods have so far been available for their study.

It is difficult to detect, and to localize at the subcellular level, the short-lived ^•^OH radical *in vivo*. There are two experimental approaches: (a) infiltration into the appropriate subcellular compartment of a membrane-impermeant ‘reporter’ that reacts with ^•^OH to give recognizable products [12]; and (b) detecting ‘collateral damage’ done to polysaccharides when cleaved by apoplastic ^•^OH *in vivo*. Membrane-impermeant probes include radioactive *N*-[^3^H]benzoyl-amide conjugates, which can be fed at tracer levels and react specifically with ^•^OH to yield tritiated water [18,24]. Non-radioactive probes can also be used in a similar way, for example D-phenylalanine (which reacts with ^•^OH to generate *o*-, *m*- and *p*-tyrosines) [14], benzoate (generating hydroxybenzoates) [25], and salicylate (producing dihydroxybenzoates) [16]. However, these non-radioactive probes may be membrane-permeant; moreover, their phenolic products are unstable in peroxidase-rich tissues, and might therefore be under-estimated. Spin labels plus ethanol have also been used, which form relatively stable ^•^OH adducts detectable by EPR spectroscopy [10,26,27]. The second approach is to detect ‘collateral damage’: unlike a glycanase or lyase, ^•^OH does not cleave a polysaccharide ‘cleanly’ by a single specific reaction (hydrolysis or β-elimination). In contrast, ^•^OH attacks polysaccharides at multiple carbons and only some of these events actually cut the polysaccharide backbone (Figure 1); concurrent reactions introduce oxo groups into the polysaccharide (‘collateral damage’) without cleaving it [12,28,29]. Thus, evidence for ^•^OH production and action in the compartment occupied by the polysaccharide of interest can be provided by a chemical ‘fingerprint’ of these oxo groups.

On attacking a polysaccharide (Figure 1), ^•^OH abstracts a carbon-bonded hydrogen atom, generating a carbon-centred radical, which, in aerobic solutions, will react with O_2_ to form an organic peroxyl radical. A hydroperoxyl radical (HO_2_^•^, usually reported as its ionized form, superoxide, O_2_^•−^) can then be eliminated, generating an aldehyde, ketone or lactone [28–30]. Abstraction of hydrogen from C-1 or C-4 of a pyranose ring quickly causes polysaccharide chain scission and may introduce an oxo group (Figure 1, reactions *c4* and *d4*); note that abstraction of H from either C-1 or C-4 may produce the same end-products because of the tautomerization reaction (↔) shown. Abstraction of H from position 5 does not inevitably cause scission, but can convert a glycosidic bond into a more labile ester bond (product of reaction *a4*) [17]. Abstraction of hydrogen from C-3 (reaction *b1*) or C-2 (or C-6 in the case of a neutral hexose residue) would introduce a relatively stable oxo group (Figure 1, grey-shaded ovals), creating a glycosulose residue without cleaving the polysaccharide chain.

**Figure 2:**
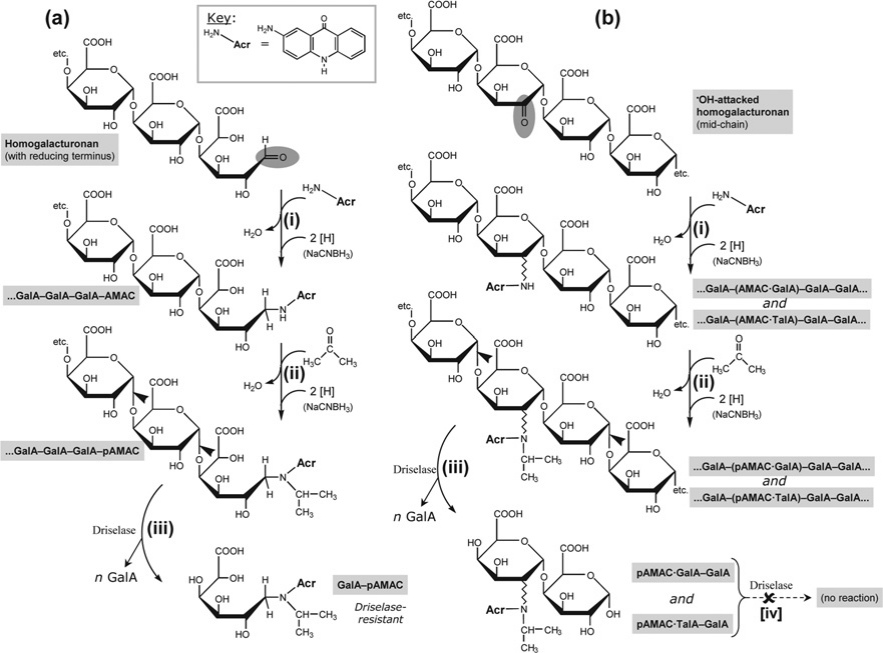
Proposed reactions of AMAC with reducing terminal and mid-chain oxo groups in homogalacturonan The oxo group to be labelled (shaded oval) is at (**a**) the polysaccharide's reducing terminus or (**b**) a mid-chain glycosulose residue created by ^•^OH attack (e.g. reaction *b3* of Figure 1). In (**b**), the oxo group could be at C-2 (as shown) or C-3 (or, in the case of a non-reducing terminal glycosulose residue, C-4). Reactions are (i) reductive amination of the oxo group with AMAC (H_2_N-Acr) plus NaCNBH_3_ to form a secondary amine, (ii) further reaction of the latter with acetone plus NaCNBH_3_ to form a more stable *N*-isopropyl tertiary amine, and (iii) digestion of the fluorescently labelled polysaccharide by Driselase (arrowheads=sites of hydrolysis). In the case of the mid-chain-labelled polysaccharide (**b**), we show in this work that the labelled disaccharide products are largely resistant to further digestion by Driselase (theoretical reaction iv). Note that in (**b**) the labelled product is a mixture of epimers. In (**a**) the abbreviation ‘GalA–pAMAC’ is not strictly correct since the carbohydrate moiety is a derivative of 6-deoxy-L-galactonic acid (formed by the reductive amination step) rather than of D-galacturonic acid; thus ‘GalA’ in our simplified nomenclature refers to the structure that was present before reaction with AMAC.

Normally, a polysaccharide is considered to possess only a single oxo group: its reducing terminus. However, ^•^OH attack introduces oxo groups indiscriminately (and thus mainly not at the reducing terminus) into sugar residues, some of which are thereby converted into glycosulose residues (Figure 1). The number of mid-chain or non-reducing terminal oxo groups would be a valuable measure of the extent of recent ^•^OH attack. Such oxo groups can be sought by radiolabelling with NaB^3^H_4_, whereby the glycosulose residue is reduced to an epimeric mixture of [^3^H]sugar residues [31], while the single oxo group of the reducing end is reduced to form a radioactive alditol. This method has been applied to detect and characterize ^•^OH attack on pectins and xyloglucans *in vitro* [17,31] and *in vivo*. As an alternative, we have now developed a method for labelling polysaccharide-bound oxo groups using the fluorescent probe 2-aminoacridone (AMAC; Figure 2). In the presence of NaCNBH_3_ (sodium cyanoborohydride), AMAC will reductively aminate oxo groups, and will therefore label the reducing terminus (Figure 2a; [32,33]) and presumably also any ^•^OH-generated oxo groups (Figure 2b). AMAC is a neutral tag at pH 8.2 [34], but at pH 3.0 it acquires a positive charge [35]. Here we describe an AMAC labelling protocol, followed by *N*-isopropylation, and its application to ^•^OH-attacked pectic polysaccharides. It is in principle applicable to the fingerprinting of naturally or artificially oxidized polysaccharides from any biological system, and to the so-far incompletely characterized products formed by polysaccharide oxidases. A recent study reports AMAC labelling for mapping glycosaminoglycan-derived disaccharides but with omission of the stabilizing *N*-isopropylation step [36].

## MATERIALS AND METHODS

### Materials

AMAC was from Fluka, tamarind xyloglucan was a generous gift from Dr K. Yamatoya (Dainippon Pharmaceutical Co.), wheat arabinoxylan was from Megazyme, and other carbohydrates were from Sigma–Aldrich. Galacturonobiose, -triose and -tetraose were prepared from homogalacturonan by partial digestion with endo-polygalacturonase and purified by gel-permeation chromatography on Bio-Gel P-2.

The Luna C_18_ HPLC column [250 mm×4.6 mm, 5 μm C_18_(2) silica 100 Å] was from Phenomenex. The HPLC eluents were from VWR or Fisher Scientific.

### ^•^OH-treatment of polysaccharides with Fenton reagent

To reduce any naturally occurring oxo groups, we pre-treated polysaccharides with NaBH_4_. In most cases, a 2% (w/v) polysaccharide solution was treated with 0.25 M NaBH_4_ in 0.5 M NaOH at 20°C for 16 h. However, pectins (0.5%, w/v) were first de-methylesterified with 0.025 M NaOH for 2 h at 0°C, and to prevent pectin precipitation we conducted the reduction reaction with 0.062 M NaBH_4_ in 0.025 M NaOH (pH 12–13).

Excess NaBH_4_ was destroyed by addition of acetic acid to pH 4.0–5.0. The sample was dialysed against water, and then treated with Fenton reagent, comprising (added in this order; final concentrations quoted): 50 mM acetate buffer (Na^+^, pH 4.5), 10 mM H_2_O_2_, 1 μM CuSO_4_ and 10 mM ascorbic acid [4]. Controls received no ascorbate, CuSO_4_ or H_2_O_2_. After 16 h at 20°C, the polysaccharide was precipitated with 75% ethanol, repeatedly washed with 75% ethanol, and air-dried.

### AMAC labelling of reducing mono- and oligo-saccharides

All AMAC work was done in subdued red light. To 0.4 μmol of dried mono- or oligosaccharide we added 4.0 μmol of AMAC in 40 μl of DMSO/acetic acid/pyridine (17:2:1), followed immediately by 40 μmol NaCNBH_3_ (freshly dissolved in 40 μl of water) and the mixture was left for 16 h at 20°C. Acetone (27 μmol) and an additional 40 μmol of NaCNBH_3_ (freshly dissolved in 40 μl of water) were then added; incubation was continued at 20°C for a further 1 h, and finally five volumes of water were added. After 5 min centrifugation at 12000 ***g***, the supernatant was purified on a mini Supelco C_18_ column, and the *N*-isopropyl-2-aminoacridone (pAMAC)-labelled product analysed by electrophoresis.

### AMAC labelling of ^•^OH-treated polysaccharides

Fenton-treated (or control) polysaccharide (150 μg, ≈0.88 μmol of sugar residues; air-dried from ethanol) was dissolved in a mixture containing 4.5 μl of 0.5% aqueous chlorobutanol, 0.5 μl of pyridine/acetic acid/water (2:2:1 by vol.) (pH ≈ 4), 0.9–1.0 μmol of AMAC in 8.9 μl of DMSO, and 12.2 μmol of NaCNBH_3_ in 6.1 μl of water. After 14–16 h at 20°C, 13.6 μmol of acetone was added and vortex-mixed, followed immediately by a further 12.2 μmol of NaCNBH_3_ in 6.1 μl of water, and incubation was continued at 20°C for 1 h. Unreacted reagents were removed by repeated washing with 75% ethanol and the precipitated polysaccharides were collected. Except in preliminary experiments, the pAMAC-labelled polysaccharides were de-lactonized, then Driselase digested for 14 days.

### De-lactonization and re-lactonization

Samples (freed of any pyridinium buffers by drying) were de-lactonized by addition of NaOH to pH >11 and incubation at 20°C for ~10 min, then adjusted with acetic acid to pH 6–7. In some experiments, GalA_2_–pAMAC was re-lactonized by addition of HCl to pH <1 and incubation at 20°C for 2 h, then neutralized with NaOH and freed of NaCl on a C_18_ column; after water-washing, pAMAC-labelled compounds were eluted either with 40 and 100% methanol or with 40 and 100% acetone. Eluates were dried and re-dissolved in pyridine/acetic acid/chlorobutanol/water (1:1:0.5:98, v/v/w/v; PyAW) before loading for electrophoresis.

### Driselase digestion

Driselase, a commercial mixture of glycanases and glycosidases from *Irpex lacteus*, was freed of insoluble matter and low-*M*_r_ solutes [37]. pAMAC-labelled poly- or oligo-saccharides were digested in 1% Driselase in PyAW at 20°C for up to 42 days. Digestion was stopped at the required time points by freezing at −20°C.

Samples were thawed, subjected to C_18_ cartridge column purification, de-lactonized and analysed by electrophoresis and/or chromatography.

### Purification of pAMAC-labelled products on a C_18_ cartridge column

The aqueous sample (typically 1–2 ml) was loaded on a 100- or 500-mg Supelco ‘Discovery’ C_18_ column (Sigma–Aldrich), which had been pre-conditioned with two volumes of methanol and rinsed with 1 or 4 ml of water. Bound pAMAC-labelled solutes were eluted with 1 or 4 ml of water followed by each of 10, 20, 30, 40, 50, 60, 70, 80, 90 and 100% methanol. Fractions were dried and redissolved in 50–200 μl of PyAW then stored at −20°C. Immediately prior to electrophoresis a portion of the sample was routinely dried, de-lactonized as above, and neutralized.

### Thin-layer chromatography

TLC was performed on Merck silica-gel plates in butan-1-ol/acetic acid/water (2:1:1). Fluorescent spots were located under a 254-nm UV lamp, and sugars were stained with thymol [38].

### High-voltage paper electrophoresis

Electrophoresis [39] was conducted on Whatman 1CHR or 3MM paper in volatile buffers at pH 2.0 (formic acid/acetic acid/water, 1:3.5:35.5 by vol.) or pH 6.5 (pyridine/acetic acid/water, 33:1:300 by vol.), routinely at 4.0 kV for 50 min. After electrophoresis the papers were dried and viewed under a 254-nm UV lamp (DocIt system with LabWorks 4.6 software; Camlab, Cambridge, UK). Non-fluorescent carbohydrates were stained with AgNO_3_ [37].

### High-pressure liquid chromatography

HPLC was conducted at 1 ml/min and room temperature on a Luna C_18_ silica column with solvents A (500 mM acetic acid, adjusted to pH 5.0 with NaOH) and B (acetonitrile). All solvent compositions are given as% B in A, by vol. The column was pre-equilibrated for 30 min with 10% B. The injected sample (20 μl) was eluted with: 0–5 min, 10% B, isocratic; 5–15 min, 10–12.5% B, linear gradient; 15–30 min, 12.5% B, isocratic; 30–35 min, 12.5–15% B, linear gradient; 35–40 min, 15% B, isocratic; 40–50 min, 15–25% B, linear gradient; 50–60 min, 25–10% B, linear gradient; 60–65 min, 10% B, isocratic. A fluorescence detector (RF 2000, Dionex) was set at *λ*_ex_ 442 nm, *λ*_em_ 520 nm.

## RESULTS

### Footnote on terminology

A compound in which a reducing sugar has been linked (via its C-1) to a pAMAC group is described here as a sugar–pAMAC conjugate, for example GalA–pAMAC (Figure 2a). In the case of a mid-chain or non-reducing terminal oxo group formed owing to prior ^•^OH attack, the corresponding products are expected to have the pAMAC group attached at a position other than C-1, as shown in Figure 2(b); we designate these product as, for example, pAMAC·GalA rather than GalA–pAMAC (Figure 2b). We use ‘UA’ to designate an unspecified uronic acid.

### Optimization of fluorescent labelling with AMAC

The fluorescent labelling technique is based on the well-known reaction of keto and aldehyde groups with AMAC + NaCNBH_3_ to give secondary amine conjugates (Figure 2, reaction i). However, this initial product may further react with additional oxo groups, as observed during reductive aminations with (NH_4_)_2_CO_3_ [40]. To produce a more stable end-product, incapable of reacting with other sugars, we treated the initially formed secondary amine with acetone + NaCNBH_3_, which is expected to generate a more stable tertiary amine containing an isopropyl group (termed pAMAC rather than AMAC labelling; Figure 2, reaction ii), and scavenge any remaining unreacted AMAC.

**Figure 3:**
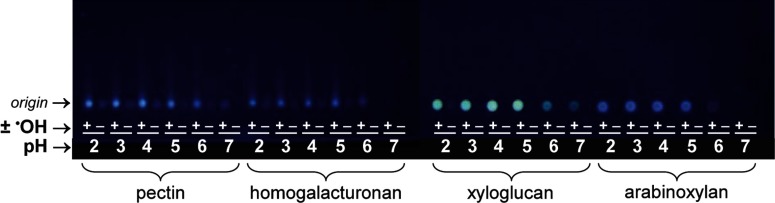
Effect of pH on AMAC labelling of polysaccharides treated with or without ^•^OH Citrus pectin, homogalacturonan, tamarind xyloglucan and wheat arabinoxylan were pre-treated with NaBH_4_, which reduced any oxo groups already present, then incubated with (+) or without (−) an ^•^OH-generating Fenton system (ascorbate + Cu^2+^ + H_2_O_2_) and finally washed by ethanol precipitation. The polysaccharides were then labelled with AMAC at pH 2.0–7.0. Unreacted AMAC was removed by ethanol washing and the polysaccharides were analysed by TLC. Fluorescence at the origin indicates polysaccharide labelling.

The reaction of an oxo group with an amine proceeds via intermediates whose formation may be catalysed by acid and/or base; in addition, some polysaccharides, e.g. homogalacturonan, form gels at low pH values. Therefore, we established a pH optimum for the AMAC labelling of several representative ^•^OH-attacked polysaccharides (Figure 3). A clear effect of the ‘Fenton treatment’ was observed, the ^•^OH-attacked polysaccharides giving a much stronger fluorescent product than the controls. A pH of 4 was chosen for routine labelling since no gelling of pectin or homogalacturonan occurred and labelling was still high (Figure 3). Labelling diminished above pH 5.

### Electrophoresis of fluorescently labelled model compounds at pH 6.5

**Figure 4:**
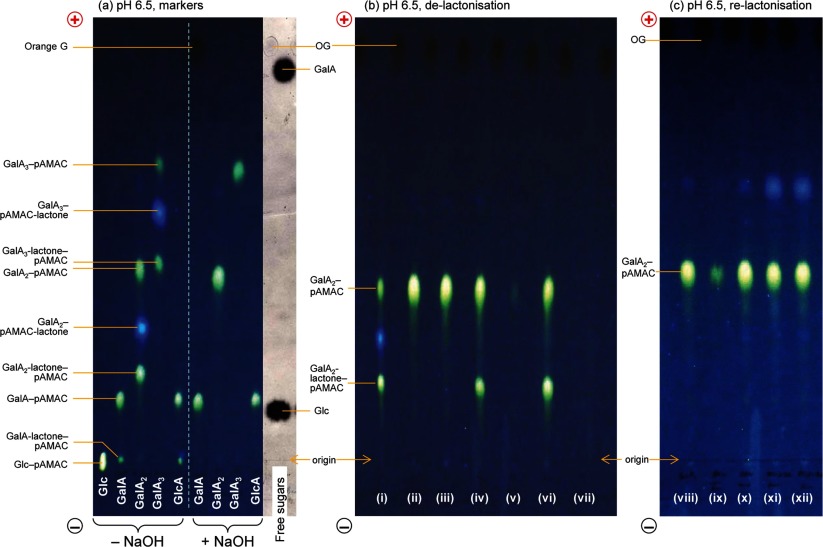
De-lactonization and re-lactonization of pAMAC-labelled oligogalacturonides (**a**) Five reducing sugars (glucose, galacturonic acid, galacturono-biose and -triose and glucuronic acid) were treated with AMAC followed by acetone. The sugar–pAMAC products were analysed by electrophoresis at pH 6.5, both directly (−NaOH) and, in the case of the acidic sugars, after de-lactonization (+NaOH). (**b**) (i) Crude GalA_2_–pAMAC (a mixture containing the fully anionic form and two putative lactones); (ii) as (i) but de-lactonized with NaOH and loaded for electrophoresis immediately after neutralization; (iii) as (i) but de-lactonized with NaOH, neutralized, stored 24 h as a solution at 4°C, and then loaded; (iv–vii) as (i) but de-lactonized with NaOH, acidified to pH <1 with HCl to promote re-lactonization, then re-isolated on C_18_ columns eluted either with (iv) 40% methanol followed by (v) 100% methanol, or with (vi) 40% acetone followed by (vii) 100% acetone. (**c**) Samples of de-lactonized GalA_2_–pAMAC were dried from various solvents, then briefly treated at pH 13 with NaOH, neutralized, and loaded for electrophoresis. (viii) Marker of authentic de-lactonized GalA_2_–pAMAC; (ix) control (in PyAW, 1:1:98 by vol., not dried); (x) dried from PyAW (1:1:98 by vol.); (xi) dried from PyAW (1:1:1 by vol.) containing 15% formic acid; (xii) dried from 15% formic acid in deionized water. The non-dried sample required large amounts of NaOH for raising the pH and therefore only a portion of it was loaded. In all cases, paper electrophoresis was at pH 6.5. Fluorescent spots were visualized under 254-nm UV. Orange G (OG) is an anionic marker (dark spot under UV). The free sugars shown in (**a**), stained with AgNO_3_ and scanned under white light, act as markers.

**Figure 5:**
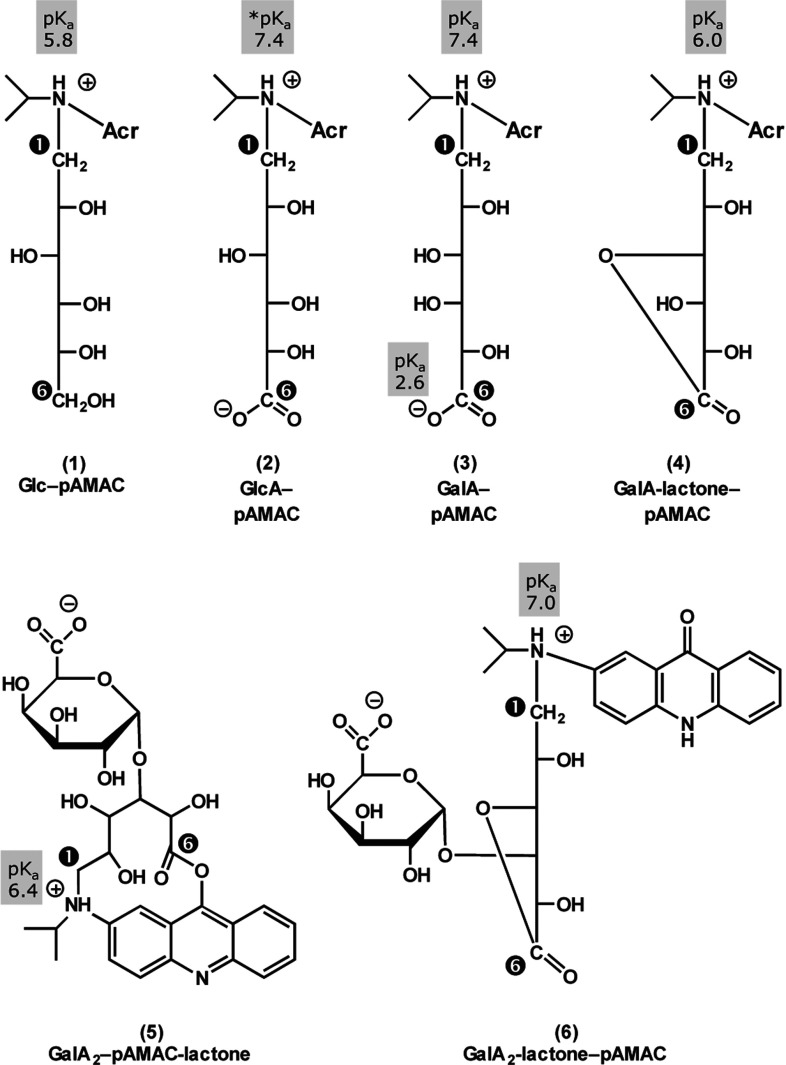
Proposed structures and estimated ionization constants of representative sugar–pAMAC conjugates Ionization constants were estimated from electrophoretic data on the basis of Offord's rules (i.e. mobility, corrected for electro-endo-osmosis, is proportional to *Q*/*M*_r_^2/3^, where *Q* is the net charge on the molecule) [41]. The full structure of ‘Acr’ is shown in structure (6). The former C-1 and C-6 of the reducing sugar moiety are marked 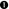 and 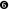 respectively.

A similar labelling method was successful with all reducing sugars tested as model compounds (neutral and acidic; mono- and oligo-saccharides). Such labelling is expected to be confined to C-1 of the reducing terminal aldose moiety (Figure 2a). In the case of neutral sugars [glucose (Figure 4a), galactose, xylose and malto-oligosaccharides (not shown)] electrophoresis at pH 6.5 showed that the major fluorescent product was slightly cationic. The measured *m*_OG_ value of Glc–pAMAC was −0.08. The observations indicate that the tertiary amino group of Glc–pAMAC (Figure 5) carries an appreciable positive charge at pH 6.5.

Offord [41] showed that electrophoretic mobility is proportional to *Q*/*M*_r_^2/3^, where *Q* is the net charge of the molecule at the pH of the electrophoresis buffer. Thus, by reference to the *m*_OG_ values of compounds with known charge and size, it is possible to estimate the charge of a novel compound of known size [38]. Using glucosamine (*M*_r_=179; *Q* ≈ +0.95 at pH 6.5) and galacturonic acid (*M*_r_=194; *Q* ≈ −1.00) for reference, we estimate that Glc–pAMAC (*M*_r_=416) has a net charge of +0.15 at pH 6.5 and thus that its tertiary amino group has a p*K*_a_ of approximately 5.8. This value is compatible with expectation on the basis that AMAC is an aniline derivative, so the effect of its amino substitution may be compared with that of the following reported p*K*_a_ values: aniline, 4.6; *N*-ethyl aniline, 5.1; *N*,*N*-diethyl aniline, 6.6 (Williams; http://research.chem.psu.edu/brpgroup/pKa_compilation.pdf). The p*K*_a_ of the amino group of free AMAC is predicted to be 4.0±0.2 (http:s://scifinder.cas.org/scifinder/), whereas acridone itself is un-ionized (the p*K*_a_ values of its O and ring-N atoms are >12 and −0.32 respectively; Williams: http://research.chem.psu.edu/brpgroup/pKa_compilation.pdf). Thus, the tertiary amino group of Glc–pAMAC (p*K*_a_ 5.8) ionizes somewhat like that of *N*,*N*-diethylaniline (p*K*_a_ 6.6).

As expected, the major pAMAC conjugates of acidic sugars (GalA, oligogalacturonides and GlcA) had a net negative charge at pH 6.5 (Figure 4a). The *m*_OG_ values of GalA–pAMAC and GlcA–pAMAC (*M*_r_=430) were typically 0.06, indicating a net charge at pH 6.5 of −0.11. Assuming that they both possess a full negative charge on the carboxy group at pH 6.5, we therefore estimate that their tertiary amino group carries a charge of +0.89, i.e. the p*K*_a_ is 7.4, much higher than in the case of Glc–pAMAC. Thus, the acidic sugar moiety strongly promotes the ionization of the amino group (Figure 5).

Similar estimates based on the observed *m*_OG_ values of GalA_2_–pAMAC, GalA_3_–pAMAC and GalA_4_–pAMAC are less reliable because the two, three or four carboxy groups will not be fully ionized at pH 6.5 [42]. The *m*_OG_ data nevertheless confirm that acidic sugar groups increase the basicity of the tertiary amino group (Figure 5).

### Electrophoresis of fluorescently labelled model compounds at pH 2.0

**Figure 6:**
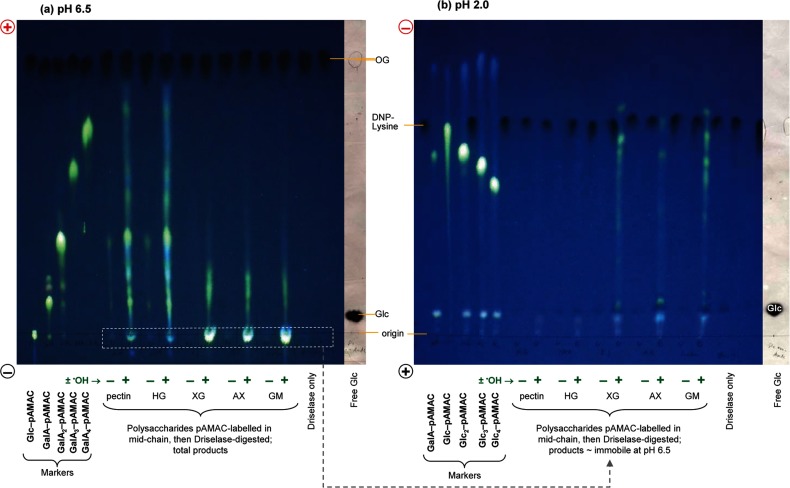
Driselase digestion products of pAMAC-labelled ^•^OH-attacked polysaccharides (**a**) The polysaccharides were pre-treated with NaBH_4_ to reduce any naturally occurring oxo groups, then treated with (+) or without (−) a ^•^OH-generating Fenton mixture, labelled with AMAC then acetone, Driselase-digested, de-lactonized and electrophoresed at pH 6.5. (**b**) pAMAC conjugates of neutral sugars, approximately co-migrating with Glc–pAMAC (dashed white rectangle), were eluted and re-electrophoresed at pH 2.0. Orange G (OG) and *N*^ε^-2,4-dinitrophenyl-lysine (DNP-Lys) are anionic and cationic markers respectively. Glc_2_–pAMAC etc. are malto-oligosaccharides labelled at the former reducing end. Polysaccharide abbreviations: HG, homogalacturonan; XG, xyloglucan; AX, arabinoxylan; GM, galactomannan.

The amino group of pAMAC conjugates will acquire a full positive charge at pH 2.0, whereas any carboxy group is only partially ionized. Accordingly, Glc–pAMAC migrated rapidly towards the cathode and the pAMAC conjugates of progressively larger neutral oligosaccharides migrated progressively more slowly (see markers in Figure 6b), as expected. Relative to the observed mobility of *N*^ε^-DNP-Lys (with one carboxy group of p*K*_a_ ≈ 2.53 and one amino group fully ionized at pH 2), the mobilities of the fluorescent conjugates indicate a net charge at pH 2 of +0.92 for Glc–pAMAC, and approximately +1.01, +1.09 and +1.07 for the labelled malto-oligosaccharides, Glc_2–4_–pAMAC, respectively. These values, close to +1, confirm that they possess a full positive charge on the tertiary amino group and no negatively charged group. The slightly lower value for Glc–pAMAC is probably due to its tendency to streak (Figure 6b).

Authentic GalA–pAMAC, oligogalacturonide–pAMAC and GlcA–pAMAC conjugates also ran as cations at pH 2.0, although slower than neutral-sugar–pAMAC conjugates of similar size: for example, GalA–pAMAC ran close to maltose–pAMAC (Figure 6b). From the mobilities and *M*_r_ values, we estimate that the carboxy group of GalA–pAMAC has the unusually low p*K*_a_ of 2.6, maybe because of the neighbouring amino group.

In conclusion, we explored the ionization properties of the pAMAC group to facilitate its use in electrophoretic characterizations. When attached to a sugar with a nearby ionizable carboxy group, the tertiary amine is considerably more basic (p*K*_a_ ≈ 7.4) than when the sugar has no ionizable carboxy group (p*K*_a_ ≈ 5.9). Thus, during electrophoresis at pH 6.5, the pAMAC fluorophore carries a partial positive charge, whose magnitude is influenced by the proximity of an ionizable carboxy group. The conjugates of acidic sugars migrate, at various rates, towards the anode. In contrast, the conjugates of neutral sugars at pH 6.5 all approximately co-migrate, with a low mobility, towards the cathode (relative to a neutral marker). At pH 2.0, pAMAC conjugates of neutral sugars have a full positive charge and no negative charge; they migrate at a speed inversely related to the size of the carbohydrate moiety (Figure 6b).

### Formation of lactones and its prevention

When a uronic acid is pAMAC-labelled at C-1, the product can be considered to be an aldonic acid derivative: 6-amino-6-deoxy-L-galactonic and 6-amino-6-deoxy-L-gulonic acids in the case of labelled D-galacturonic and D-glucuronic acids respectively (Figure 5). Many aldonic acids are prone to lactonize in slightly acidic aqueous solution. Indeed, when glucuronic acid and galacturono-biose and -triose were pAMAC-labelled and subjected to electrophoresis at pH 6.5 (without a de-lactonization step), each migrated as three spots (Figure 4a). Two of these fluoresced with the yellow–green colour expected of pAMAC conjugates, while the middle spot fluoresced blue.

The two slower-migrating spots could be converted into the fastest-migrating one by a brief treatment at pH >11 (Figure 4a), which cleaves lactone rings. The slower-migrating yellow–green-fluorescing spot, but not the blue spot, was partially regenerated if the de-lactonized sample was treated at pH <1 (Figure 4b: iv, vi), which promotes re-lactonization. These results indicate that the slowest-migrating spot is a lactone of the fastest-migrating spot.

In conjugates of acidic monomers (GalA–pAMAC and GlcA–pAMAC), the yellow–green-fluorescing lactone approximately co-migrated with Glc–pAMAC. Using the approach outlined for Glc–pAMAC, we estimate that the p*K*_a_ of the tertiary amino group in GalA-lactone–pAMAC is 6.0. Thus the conjugates of monomer lactones resemble those of glucose (both having no anionic group).

It might be expected that GalA–pAMAC would have a higher *m*_OG_ value than GalA_2_-lactone–pAMAC since they both have a single ionizable carboxy group whereas the latter is larger, giving it a smaller *Q*/*M*_r_^2/3^ value. However, the opposite was observed, showing that the presence of an ionizable carboxy group near to the amino group helps the latter to acquire a positive charge (p*K*_a_=7.4) more effectively does the more distant ionizable carboxy group of GalA_2_-lactone–pAMAC (estimated p*K*_a_=7.0; Figure 5).

The blue-fluorescing middle spots may be lactones involving the aromatic O atom of the pAMAC moiety [Figure 5, structure (5)], designated a ‘pAMAC-lactone’ conjugate, whose colour of fluorescence would thereby be altered. In accordance with this interpretation, neutral sugars never yielded blue-fluorescing products. In the case of GalA_2_–pAMAC-lactone, and assuming that it has one full negative charge and a partial positive charge, we estimate the p*K*_a_ of its amino group to be 6.4 [Figure 5, structure (5)].

Treatment of pAMAC-labelled oligogalacturonides with formic acid produced an additional blue-fluorescing spot that migrates faster than both the yellow–green-fluorescing spots (Figure 4c: xi, xii). Formic acid, which is often used to terminate Driselase digestions, was therefore avoided.

In conclusion, we found that reducing-end-labelled uronic acids (e.g. GalA_2_–pAMAC) can form two different lactones and thus give rise to three fluorescent spots. This complication was solved by de-lactonization with a brief NaOH treatment, giving a single fluorescent spot. The blue- and yellow–green-fluorescing lactones were probably structures (5) and (6) respectively (Figure 5).

AMAC-labelling has also been used to detect mono- and oligo-saccharides during fluorophore-assisted carbohydrate electrophoresis (FACE) [43], which in some cases has given rise to inexplicable spots. No alkali treatment was used, and the unknown spots could include lactones of acidic AMAC-labelled sugars [33].

### Driselase digestion of reducing-end-labelled oligosaccharides

With few exceptions, Driselase can digest plant cell wall polysaccharides by the concerted action of glycanases and glycosidases to release monosaccharides [39]. Often, glycosidases are highly specific for the non-reducing terminal sugar residue attacked but are relatively unaffected by the nature of the aglycone to which it is attached [1]. We therefore predicted that oligosaccharide–pAMAC conjugates would be progressively digested by Driselase to yield free monosaccharides plus a fluorescent monosaccharide–pAMAC conjugate.

**Figure 7:**
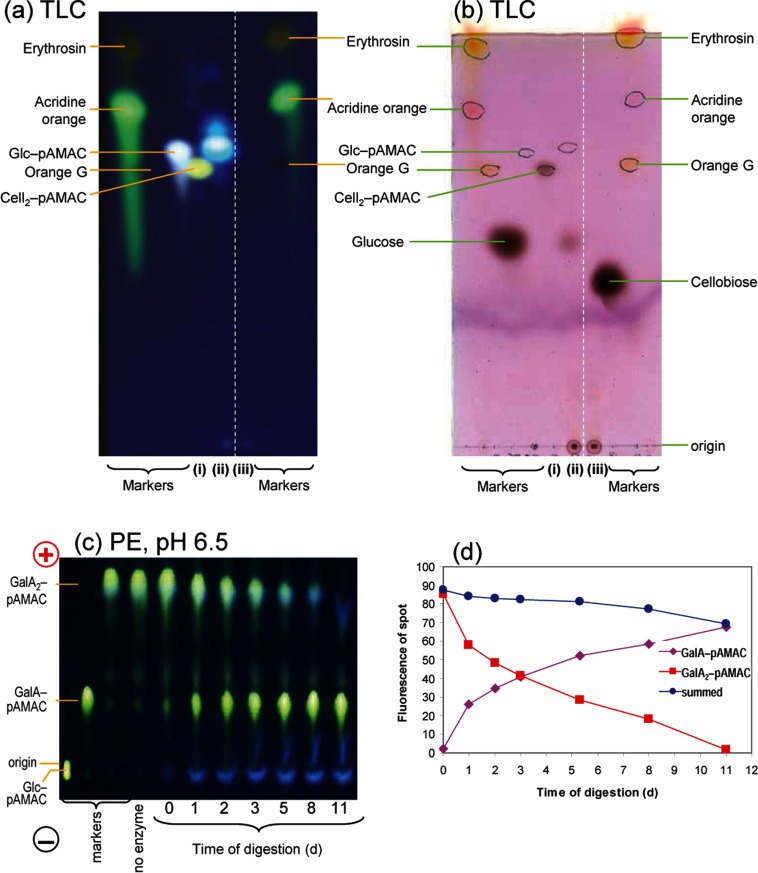
Driselase digestion of reducing-end-labelled cellobiose and galacturonobiose (**a** and **b**) TLC of cellobiose–pAMAC (Cell_2_–pAMAC) (i) alone and (ii) after digestion with Driselase for 3 days; (iii) Driselase alone. (**a**) UV fluorescence, (**b**) same TLC plate after sugar-staining with thymol-H_2_SO_4_, viewed under visible light. (**c** and **d**) Paper electrophoresis at pH 6.5 of galacturonobiose–pAMAC (GalA_2_–pAMAC) digested with Driselase for 0–11 days. (**c**) Viewed under a 254-nm UV lamp. (**d**) Quantification of fluorescence in starting material and product (arbitrary units of fluorescence; DocIt software).

Driselase indeed digested cellobiose–pAMAC to fluorescent Glc–pAMAC (Figure 7a) plus free glucose (Figure 7b), essentially complete within 3 days. A similar experiment on GalA_2_–pAMAC showed a comparable result (Figure 7c), although the hydrolysis took ~2 weeks to approach completion (half-life ≈ 2–3 days) (Figure 7d). Thus, as expected, prolonged Driselase digestion will release monosaccharide–pAMAC conjugates from reducing-end-labelled oligosaccharides, and therefore also plant cell-wall polysaccharides.

**Figure 8:**
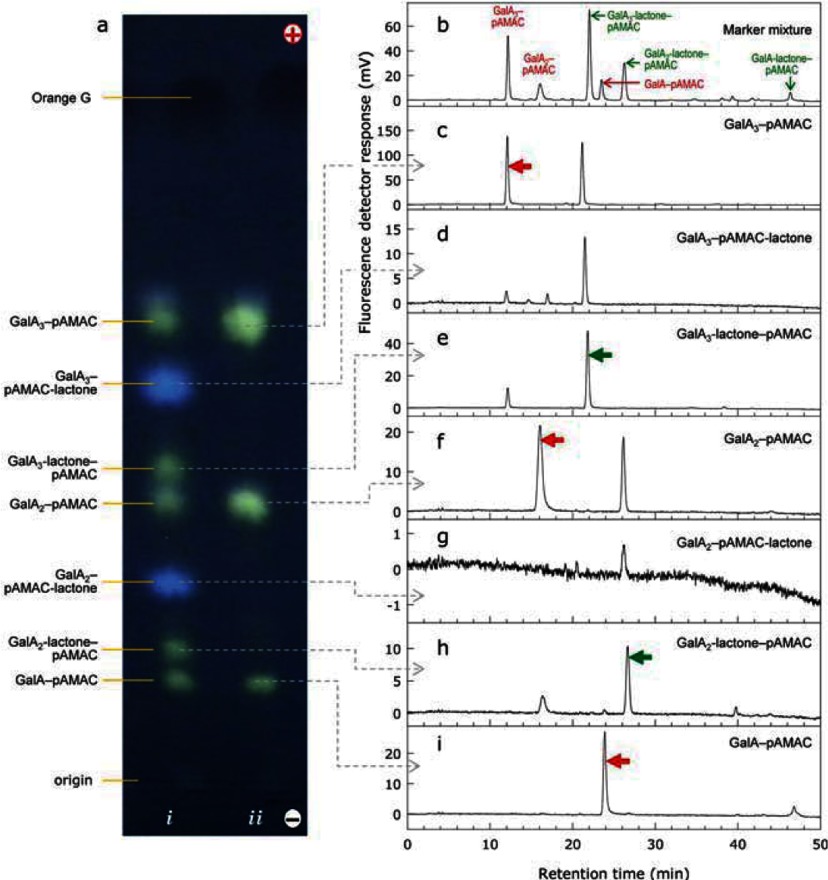
HPLC resolution of individual GalA*_n_*–pAMAC conjugates eluted from an electrophoretogram (**a**) High-voltage paper electrophoretogram of GalA*_n_*–pAMAC conjugate mixtures similar to that in Figure 4(a). *i*, Not de-lactonized; *ii*, de-lactonized. (**b**) HPLC profile on a Luna C_18_ column of the sample loaded in (**a**) *i*. (**c**–**i**) HPLC profiles of individual spots eluted from the electrophoretogram as shown by broken arrows. In each case the peak corresponding to the named compound is indicated with an arrowhead.

HPLC was used as an additional analytical approach. The UA–pAMAC markers and their respective yellow–green-fluorescing lactones were eluted from electrophoretograms. All were well resolved from each other by HPLC (Figure 8). Some acid ↔ lactone inter-conversion occurred between electrophoresis and the HPLC step (Figure 8c–8i), but if a mixture of GalA*_n_*–pAMAC conjugates (Supplementary Figure S1a at http://www.biochemj.org/bj/463/bj4630225add.htm) was freshly de-lactonized and then immediately subjected to HPLC, only the acid peaks were observed (Supplementary Figure S1b). The elution sequence was: largest first; and acid before lactone. Thus, as expected, elution was in the order of decreasing polarity. The blue-fluorescing spots, when isolated by electrophoresis, co-eluted on the HPLC with the corresponding yellow–green-fluorescing lactones.

### Driselase digestion of five mid-chain-labelled ^•^OH-attacked polysaccharides

^•^OH attack is expected to introduce oxo groups rather indiscriminately along a polysaccharide chain, thus mostly not at the reducing terminus. The fluorescent products that are created when these oxo groups are pAMAC-labelled do not necessarily indicate new termini introduced by polysaccharide scission, but represent oxo groups that are among the products formed by ‘collateral damage’ when ^•^OH attacks polysaccharides in an aerobic environment [29]. The method for labelling mid-chain or non-reducing terminal oxo groups was tested on ^•^OH-treated and control samples of five polysaccharides. Before exposure to ^•^OH, the polysaccharides had been treated with NaBH_4_, which reduces any existing oxo groups (including the reducing ends) to unreactive alcohols. After the ^•^OH treatment, the polysaccharides were reacted with AMAC/NaCNBH_3_ followed by acetone/NaCNBH_3_, the high-*M*_r_ products were exhaustively digested with Driselase, and the digestion products were de-lactonized and analysed by electrophoresis (Figure 6a).

Electrophoresis at pH 6.5 revealed fluorescent anionic pAMAC conjugates in all five cases (Figure 6a). The anionic conjugates generated from the two acidic polysaccharides (pectin and homogalacturonan) were numerous and abundant, including spots that appeared to be acidic mono- and di-saccharides approximately co-migrating with GalA–pAMAC and GalA_2_–pAMAC respectively. The anionic pAMAC conjugates were fewer and fainter in the case of the three neutral polysaccharides tested (xyloglucan, arabinoxylan and galactomannan). Nevertheless, ^•^OH-untreated polysaccharides gave only very faint fluorescent spots, showing that the observed fluorescent products were ‘fingerprint’ compounds, useful for recognizing ^•^OH-attacked polysaccharides. The anionic products from neutral polysaccharides probably indicate the hydrolysis of esters and lactones that were introduced (Figure 1, reactions *a4* and *c4*) by ^•^OH, which converts some glycosidic bonds into ester bonds [31].

The slightly cationic fluorescent pAMAC conjugates of neutral sugars (co-migrating with Glc–pAMAC) obtained from the five polysaccharides were eluted from the pH 6.5 electrophoretogram (Figure 6a; broken white rectangle), and re-electrophoresed at pH 2.0 (Figure 6b). In the case of the three neutral polysaccharides, a series of cationic products was resolved (Figure 6b). Because of their behaviour at pH 6.5, they must represent pAMAC-conjugates of neutral sugars, which, from their electrophoretic mobilities at pH 2.0, we estimate to include di- to hexa-saccharides.

We conclude that pAMAC labelling is an excellent means of recognizing polysaccharides that have been attacked by ^•^OH.

### Further analysis of Driselase digestion products of pAMAC·pectin

**Figure 9:**
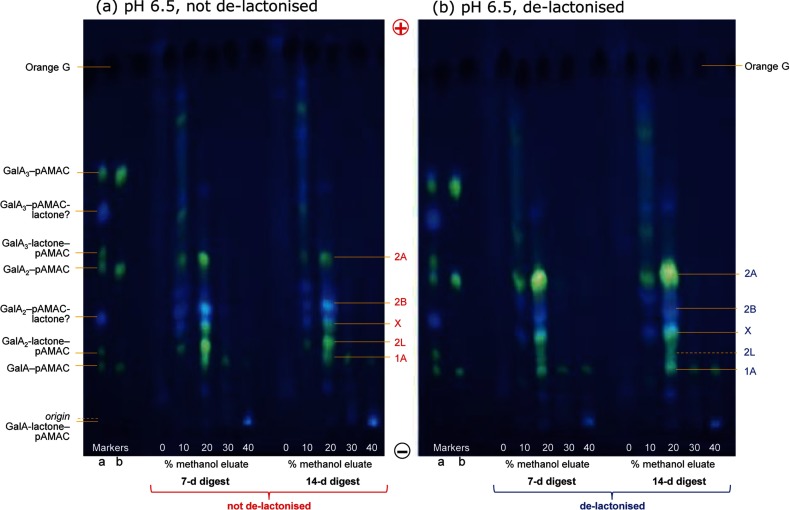
Driselase digestion products of pAMAC-labelled ^•^OH-attacked pectin: electrophoretic analysis (**a**) De-esterified pre-reduced citrus pectin was successively treated with ^•^OH, AMAC, acetone and finally Driselase for 7 and 14 days; the products were then fractionated on a mini C_18_ column eluted with 0–40% methanol. The image shows electrophoretograms at pH 6.5 of the fluorescent fractions. (**b**) As in (**a**) but each fraction was de-lactonized before electrophoresis. Markers a and b are identical mixtures before and after de-lactonization.

The products of ^•^OH attack on an acidic polysaccharide were analysed in more detail in the case of citrus pectin. The Driselase-generated anionic products of pAMAC·pectin were eluted from a C_18_ cartridge, with most of the pAMAC-labelled products eluting in 10% or 20% methanol (Figure 9). There was little difference between the 7- and 14-day digests, indicating that the formation of Driselase-stable products was essentially complete within 7 days.

When the Driselase-digested sample was not de-lactonized, four major yellow–green-fluorescing spots (1A, 2L, X and 2A) were seen (Figure 9a). In addition, a blue-fluorescing spot (2B) migrated faster than X. When the sample was freshly de-lactonized before electrophoresis, 1A was unchanged, 2L and 2B decreased slightly in intensity, and X and 2A became more intense (Figure 9b).

2A, the predominant spot after de-lactonization, is proposed to be a pAMAC·UA_2_ conjugate (e.g. Figure 2b, reaction iii) as it co-migrated with GalA_2_–pAMAC, which is expected to have a similar *m*/*z* ratio. Spot 2A is clearly not GalA_2_–pAMAC itself (a disaccharide with pAMAC at the former reducing terminus) because this would have been digested by Driselase to GalA–pAMAC plus free GalA within 7–14 days (Figure 7d). 2A is thus proposed to be the most useful chemical ‘fingerprint’ of ^•^OH attack on pectin.

Spot 1A is proposed to be a UA–pAMAC conjugate as it was released by Driselase and co-migrated with authentic marker GalA–pAMAC. The formation of UA–pAMAC probably occurred after ^•^OH had attacked at C-4 thus creating a new reducing terminus (Figure 1, reaction *d4*).

2L co-migrated with GalA_2_-lactone–pAMAC, and was at least partially de-lactonized by NaOH (Figure 9); however, unlike GalA_2_-lactone–pAMAC itself (Figure 4b), some of the 2L remained (or rapidly re-formed) after NaOH treatment, producing a streak on the electrophoretogram. Therefore, spot 2L is proposed to contain pAMAC·UA_2_-lactone(s) that can be de-lactonized to form 2A.

X migrated faster than authentic GalA_2_-lactone–pAMAC (Figure 9) and is not a lactone because it increased in intensity after de-lactonization; it is possibly a pAMAC·UA_2_ with one of the UA residues having a higher p*K*_a_ than GalA. Since the intensity of X increased after the mixture had been de-lactonized, we conclude that a spot migrating slower than X contains a lactone form of X.

In addition to these four yellow–green-fluorescing compounds, there was a blue-fluorescing spot, 2B, that approximately co-migrated with GalA_2_–pAMAC-lactone (Figure 9a). The blue fluorescence suggests lactonization involving the aromatic O atom, as proposed for GalA_2_–pAMAC-lactone [Figure 5, structure (5)]. However, 2B decreased only slightly (or transiently) upon de-lactonization, unlike GalA_2_–pAMAC-lactone, which disappeared completely (Figure 4b). Therefore we propose that 2B is different from GalA_2_–pAMAC-lactone and that, like de-lactonized 2L, de-lactonized 2B has a propensity to re-lactonize (probably to 2L) when neutralized.

HPLC was also investigated. Pectin was ^•^OH-treated, pAMAC-labelled and Driselase-digested, as before. The products were eluted from a C_18_ cartridge with 20% methanol and (without de-lactonization) analysed by HPLC (Supplementary Figure S1c). The HPLC profile broadly resembled that produced by a mixture of GalA*_n_*–pAMAC conjugates (Supplementary Figure S1a), except that, as expected, no trisaccharide conjugates were observed since Driselase can readily hydrolyse such large pectic fragments. Peaks were tentatively assigned on the basis of similarity to end-labelled compounds with similar overall constitution (1A ≈ GalA–pAMAC, 2A ≈ GalA_2_–pAMAC, 2L ≈ GalA_2_-lactone–pAMAC). In addition, the pAMAC·UA*_n_* products included at least eight unidentified compounds, one of which (retention time ≈ 9.5 min) was a major component and is therefore proposed to be the major fluorescent compound marked as ‘X’ in Figure 8. Although peaks were observed that approximately co-eluted with GalA_2_–pAMAC and GalA_2_-lactone–pAMAC, these cannot be their true identities because they would have been hydrolysed to GalA–pAMAC by the prolonged Driselase treatment applied. Furthermore, the putative 2L peak which upon HPLC co-elutes with GalA_2_-lactone–pAMAC failed to disappear after de-lactonization, unlike true GalA_2_-lactone–pAMAC (Supplementary Figure S1b). Thus, the Driselase-resistant disaccharide-based product(s), some of which cannot be stably de-lactonized, as well as the early-eluting peak ‘X’, can be taken as HPLC peaks diagnostic of ^•^OH-attacked pectin, providing another fingerprinting strategy as an alternative to electrophoresis.

**Figure 10:**
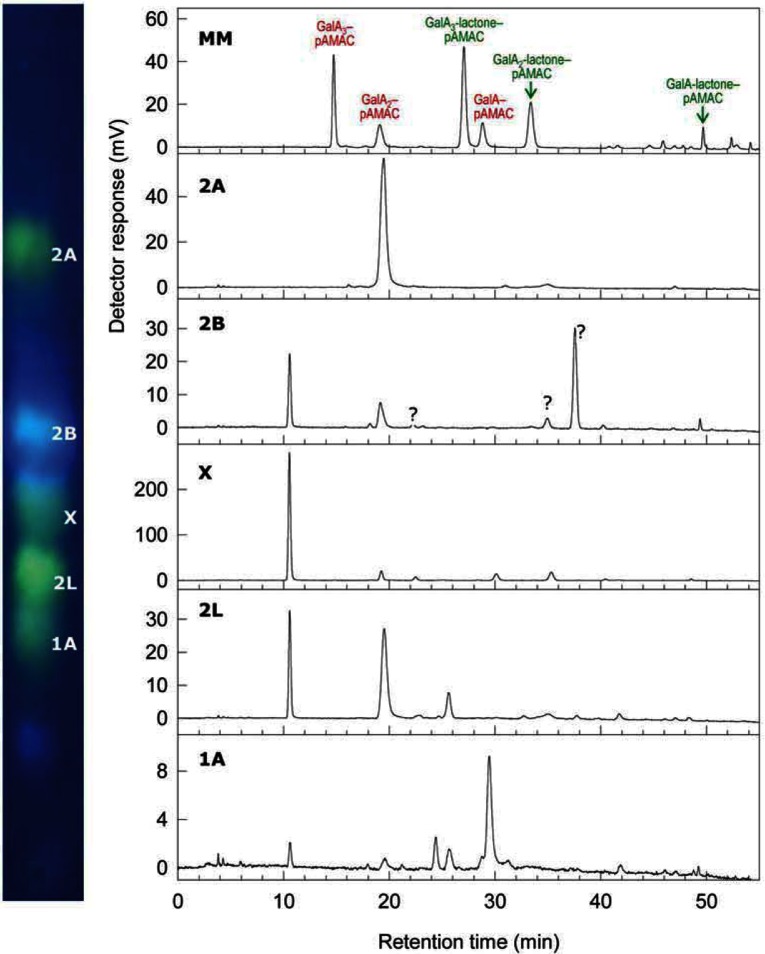
Driselase digestion products of pAMAC-labelled ^•^OH-attacked pectin: HPLC analysis Pectin was ^•^OH-treated, processed and electrophoresed as in Figure 9. The five indicated spots, putative pAMAC·UA*_n_* conjugates, were eluted and analysed by HPLC. MM=marker mixture of GalA*_n_*–pAMAC conjugates.

To test the correspondence between electrophoresis spots and HPLC peaks, we eluted each fluorescent spot (1A, 2L, X, 2A and 2B) from an electrophoretogram (as in Figure 8) and ran it on HPLC (Figure 10). 2A, X and 1A gave peaks (Figures 10b, d and f) matching their expected retention times (Supplementary Figure S1c). On the other hand, several peaks were resolved from the blue-fluorescing compound 2B (Figure 10c), at least three of which matched unidentified peaks in Supplementary Figure S1c. Spot 2L gave a main peak co-eluting with GalA_2_–pAMAC (showing acid ↔ lactone inter-conversion) and an additional major peak matching the retention time of X. Thus, 2L was (or contained) a lactone of X, electrophoresing slightly slower than the X.

In conclusion, it is important to distinguish labelling of mid-chain or non-reducing terminal glycosulose residues (*b3* and *d4* respectively; Figure 1), from that at new reducing termini, created by hydrolysis or β-elimination of glycosidic bonds or by ^•^OH-initiated reactions (*d4*; Figure 1). Indeed, in future applications of the labelling technique *in vivo*, both glycanase and lyase action (introducing new reducing termini) and ^•^OH attack may well be occurring simultaneously; Driselase distinguishes these alternatives. In plant cell-wall polysaccharides, the pAMAC-labelled reducing-end sugar will, according to known patterns of Driselase action [37], be released as a fluorescent monosaccharide-derivative, e.g. GalA–pAMAC (Figure 2a, reaction iii). In contrast, it was not known whether the glycosidic bond of a mid-chain or non-reducing terminal pAMAC·UA residue would be recognized as a Driselase substrate to yield a fluorescent monosaccharide (pAMAC·UA) or resist digestion and thus give a pAMAC·disaccharide (Figure 2b, reaction iii).

Our data support the latter possibility. In pectin, the most clearly distinguishing product serving to ‘fingerprint’ mid-chain or non-reducing terminal oxo groups was 2A (Figure 9). Although co-electrophoresing with GalA_2_–pAMAC and thus likely to possess an identical *m*/*z* ratio, product 2A is clearly a different substance characterized by its resistance to Driselase. In addition, the two probable lactone forms of 2A (i.e. 2L and 2B, suggested to be pAMAC·UA_2_-lactone and pAMAC-lactone·UA_2_ respectively) differ from the corresponding reducing-end-labelled compounds (GalA_2_-lactone–pAMAC and GalA_2_–pAMAC-lactone) by the fact that they cannot be stably de-lactonized by NaOH.

On theoretical grounds (Figure 2b), spot 2A could comprise several closely related compounds, including 2-*O*-pAMAC·GalA-α-(1→4)-GalA, 3-*O*-pAMAC·GalA-α-(1→4)-GalA, 4-*O*-pAMAC·GalA-α-(1→4)-GalA, 2-*O*-pAMAC·TalA-α-(1→4)-GalA, 3-*O*-pAMAC·GulA-α-(1→4)-GalA and 4-*O*-pAMAC·GlcA-α-(1→4)-GalA, depending on which carbon atom was initially attacked by the ^•^OH and whether the reductive amination maintained or reversed the epimerism. It is likely that all six compounds would resist digestion by Driselase because the pAMAC substitution prevents α-galacturonidase action and because Driselase probably lacks α-D-taluronidase, α-D-guluronidase and α-D-glucuronidase.

### Conclusion

This paper reports a simple and effective means of fingerprinting polysaccharides to reveal evidence of mid-chain and non-reducing terminal oxo groups (of glycosulose residues) diagnostic of ^•^OH attack. The major disaccharide-based products are different from those generated by newly formed reducing termini. Thus, of the three major types of reaction mechanism potentially capable of causing mid-chain scission of a polysaccharide (^•^OH attack, endo-hydrolysis and β-elimination), we have developed a novel procedure for specifically documenting the occurrence of ^•^OH attack.

Cell walls often contain (besides polysaccharides) phenolics and glycoproteins, which may possibly also be attacked by ^•^OH and then pAMAC-labelled. Using polysaccharide-specific enzymes should restrict the observed low-*M*_r_ fingerprint products to those derived from the polymers of interest. Naturally occurring low-*M*_r_ apoplastic antioxidants would be unlikely to interfere in the labelling of polysaccharide-bound oxo groups since such compounds (e.g. ascorbate and flavonoids) are not known to reduce oxo groups, and would be washed out during AIR preparation. We are currently applying the procedure to study polysaccharide modifications occurring during the softening process in ripening fruit.

With substitution of other hydrolytic enzyme preparations in place of Driselase, the methodology can in principle be applied to polysaccharide and other potential polymeric ^•^OH targets isolated from a wide range of animal, plant and microbial sources in which interesting cases of polysaccharide cleavage are thought to be occurring. It is also predicted to be a valuable method for characterizing the action of the recently discovered polysaccharide oxidases: in this case, Driselase itself should be a suitable enzyme preparation for fragmenting the labelled polysaccharides since much of the work is focused on plant cell-wall-derived biomass [3].

## Online data

Supplementary data
