# Supplementary material for: Fingerprinting of hydroxyl radical-attacked polysaccharides by N-isopropyl-2-aminoacridone labelling
Source: Biochem J. 2014 Sep 22;463(Pt 2):225–37. doi: 10.1042/BJ20140678 (PMC4170706; doi:10.1042/BJ20140678)
Supplement: Supplementary data [file bj4630225add.pdf]

Biochem. J. (2014) **463**, 225–237 (Printed in Great Britain) doi:10.1042/BJ20140678

SUPPLEMENTARY ONLINE DATA

Fingerprinting of hydroxyl radical-attacked polysaccharides by *N*-isopropyl-2-aminoacridone labelling

Robert A. M. VREEBURG*[1], Othman B. AIRIANAH*[2] and Stephen C. FRY*[3]

*The Edinburgh Cell Wall Group, Institute of Molecular Plant Sciences, School of Biological Sciences, Daniel Rutherford Building, The King's Buildings, Edinburgh EH9 3JH, U.K.

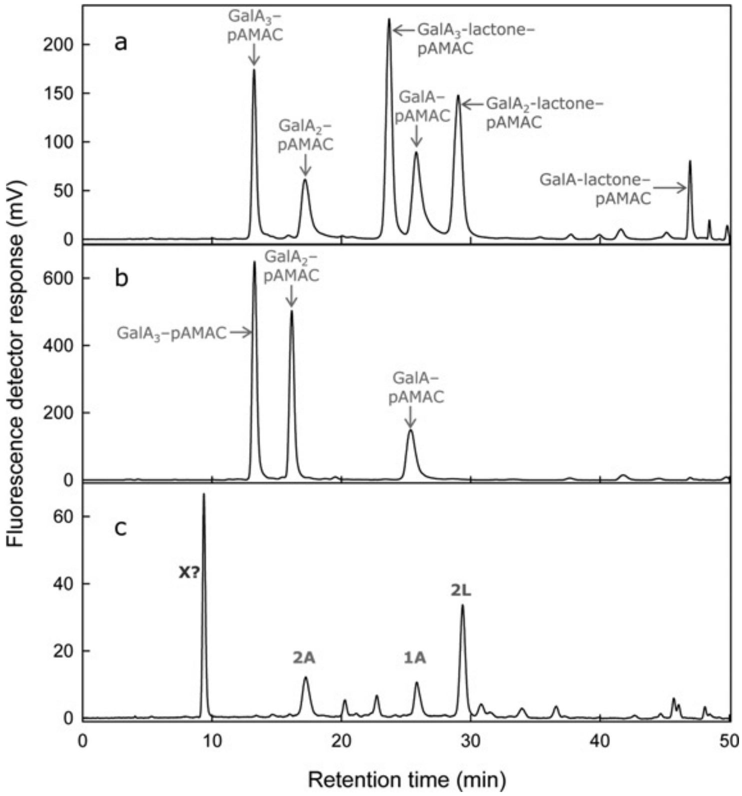

Figure S1 HPLC of Driselase digestion products of pectin, pAMAC-labelled after •OH-attack, compared with UA–pAMAC conjugates

(**a**) Mixture of $GalA_n$–pAMAC conjugates similar to that in Figure 4(a) of the main text. (**b**) As in (**a**) but de-lactonized, showing only the free acids. (**c**) •OH-attacked pectin was pAMAC-labelled and Driselase-digested, and the fluorescent products were enriched by use of a C_{18} cartridge. The mixture of products in the 20 % methanol eluate (not de-lactonized) was then subjected to HPLC on a Luna C_{18} column. Peaks in (**c**) are labelled 1A, 2A and 2L on the basis of the similarity of their HPLC retention times relative to those of reducing-end-labelled compounds that are identical (=) or have equivalent constitution (≡): 1A = GalA–pAMAC; 2A ≡ $GalA_2$–pAMAC; 2L ≡ $GalA_2$-lactone–pAMAC.

Received 29 May 2014/28 July 2014; accepted 29 July 2014
Published as BJ Immediate Publication 29 July 2014, doi:10.1042/BJ20140678

[1] Present address: NAK, P.O. Box 1115, 8300 BC Emmeloord, The Netherlands
[2] Present address: School of Biosciences & Biotechnology, Faculty of Science & Technology, The National University of Malaysia, 43600 Bangi, Selangor, Malaysia.
[3] To whom correspondence should be addressed (email S.Fry@ed.ac.uk).

© 2014 The Author(s) This is an Open Access article distributed under the terms of the Creative Commons Attribution Licence (CC-BY) (http://creativecommons.org/licenses/by/3.0/) which permits unrestricted use, distribution and reproduction in any medium, provided the original work is properly cited.

Biochemical Journal www.biochemj.org
